# Comparative meta-omics for identifying pathogens associated with prosthetic joint infection

**DOI:** 10.1038/s41598-021-02505-7

**Published:** 2021-12-09

**Authors:** Karan Goswami, Alexander J. Shope, Vasily Tokarev, Justin R. Wright, Lavinia V. Unverdorben, Truc Ly, Jeremy Chen See, Christopher J. McLimans, Hoi Tong Wong, Lauren Lock, Samuel Clarkson, Javad Parvizi, Regina Lamendella

**Affiliations:** 1 Rothman Institute, Philadelphia, PA USA; 2 Contamination Source Identification LLC, Huntingdon, PA USA

**Keywords:** Computational biology and bioinformatics, Microbiology, Biomarkers, Health care, Medical research

## Abstract

Prosthetic joint infections (PJI) are economically and personally costly, and their incidence has been increasing in the United States. Herein, we compared 16S rRNA amplicon sequencing (16S), shotgun metagenomics (MG) and metatranscriptomics (MT) in identifying pathogens causing PJI. Samples were collected from 30 patients, including 10 patients undergoing revision arthroplasty for infection, 10 patients receiving revision for aseptic failure, and 10 patients undergoing primary total joint arthroplasty. Synovial fluid and peripheral blood samples from the patients were obtained at time of surgery. Analysis revealed distinct microbial communities between primary, aseptic, and infected samples using MG, MT, (PERMANOVA p = 0.001), and 16S sequencing (PERMANOVA p < 0.01). MG and MT had higher concordance with culture (83%) compared to 0% concordance of 16S results. Supervised learning methods revealed MT datasets most clearly differentiated infected, primary, and aseptic sample groups. MT data also revealed more antibiotic resistance genes, with improved concordance results compared to MG. These data suggest that a differential and underlying microbial ecology exists within uninfected and infected joints. This study represents the first application of RNA-based sequencing (MT). Further work on larger cohorts will provide opportunities to employ deep learning approaches to improve accuracy, predictive power, and clinical utility.

## Introduction

Next-generation sequencing (NGS) technologies have enabled billions of nucleic acid fragments to be sequenced in a high-throughput manner. Recently, NGS technologies, including shotgun metagenomics (MG), have been applied to clinical microbiological testing, allowing for a comprehensive approach to pathogen detection in various clinical specimens^1,2^. In contrast to PCR-based methods, untargeted methods (MG, MT) randomly sequence DNA and RNA directly^3^. Improvements in sequencing throughput, library preparation methods, and human nucleic acid removal strategies have accelerated the application of shotgun meta-omics methods for infectious disease detection^4–6^. In particular, MT analyses generate gene expression data that can reveal active pathways, offering an advantage over MG, which doesn’t differentiate between active and inactive genes in a sample. Thus, in clinically-relevant specimens, RNA expression allows for identification of active pathogenic organisms, active virulence mechanisms, and functional antimicrobial-resistance mechanisms^7,8^.

Prosthetic joint infection (PJI) complicates 1–2% of total hip (THA) and knee (TKA) arthroplasty, and despite considerable efforts, rates do not appear to be decreasing^9,10^. PJI incurs a substantial burden, as it often requires additional revision procedures and hospital stays, ultimately increasing risk of patient mortality^11^. When pathogenic organisms cannot be isolated, culture-negative PJI (CN-PJI), the likelihood of treatment success falls below 70%, leaving patients at risk of complications^12^. The rate of CN-PJI is reportedly between 7 and 15%, but rates as high as 42% have been reported^13,14^.

Routine microbiological culture is the standard for identifying microorganisms, but PJI culture sensitivity remains low, ranging from 39 to 70%^12^, incentivizing molecular diagnostic techniques (PCR, NGS). Amplicon NGS methods, like 16S rRNA gene sequencing have demonstrated success in identifying causative organisms in culture-negative infections, but there are significant shortcomings like PCR biases and limited taxonomic resolution^15–17^. Shotgun sequencing circumvents culture and PCR-based limitations by enabling a comprehensive view of the identity and functional gene content of microbial consortia populating a clinical specimen^18^. Metagenomics analysis has previously shown high concordance with culture results for diagnosing PJIs^19^; however, to the best of our knowledge, no work has yet been done comparing metatranscriptomics results to culture results in this context.

Our goal was to compare 3 NGS techniques (16S, MG, and MT) in their ability to distinguish microbial profiles in synovial fluid and blood from patients undergoing primary, aseptic revision, and revision for PJI. Additionally, we assessed concordance of these NGS techniques to culture-based methods. We hypothesized that untargeted techniques, including MG and MT would outperform 16S amplicon sequencing taxonomically resolving and differentiating microbial profiles associated with PJIs. Additionally, we wanted to evaluate the utility of shotgun MG and MT methods for detecting antimicrobial resistance genes in clinical specimens.

## Methods

### Statement of ethical approval

This study was approved by the internal review board of Thomas Jefferson University (IRB protocol 17D.059). All patients in this study provided informed consent prior to surgical procedures either during pre-operative office visits or in the pre-operative area. Study methods were performed in accordance with approved guidelines.

### Study cohorts and patient classification

The study cohort consisted of a total of 30 patients, with a mean age of 68.1 years (range 53–85) undergoing knee or hip surgery in a single institution between September 2018 to January 2019. The study samples were subdivided into 3 groups, patients undergoing primary arthroplasty (native joints), patients with aseptic failure, and patients with PJI. Individuals were excluded from the study if they were under the age of 18, had a body mass index (BMI) greater than 40, or had a diagnosis of diabetes with a current HbA1c > 8. Patients with primary joints had a diagnosis of osteoarthritis in either the knee or hip and were receiving a TKA or THA. Patients were excluded from the primary arthroplasty group if they had any previous surgical procedure on the operative joint, had received a corticosteroid or viscosupplement injection within 9 months of the procedure, or showed any signs of infection or osteoarthritis in the contralateral joint. Patients with infected joints had undergone a TKA or THA and presented with signs of infection and met the ICM criteria for a PJI^20^. Patients were assigned aseptic revision group if they were undergoing revision arthroplasty but did not meet the ICM criteria for PJI. Patient demographics, cohorts, and procedure information are in Table 1. Applicable physical exam, clinical laboratory results, and ICM PJI classification of patients are provided in Supplementary Table S1.

**Table 1:** Patient characteristics and cohorts.

Sample ID	Cohort	Sex	Age	Procedure
4	Primary	F	60	R TKA
5	Primary	F	63	R TKA
6	Primary	F	53	R TKA
7	Primary	M	67	R TKA
8	Primary	F	84	L TKA
10	Primary	M	64	B/L; L TKA
26	Primary	F	67	R TKA
27	Primary	M	60	R TKA
33	Primary	M	72	R TKA
34	Primary	F	85	L TKA
12	Aseptic	F	70	R knee aspirate
16	Aseptic	M	70	L TKR 1-STAGE
22	Aseptic	F	78	R TKR 1-stage
24	Aseptic	F	57	R THR 1-stage
25	Aseptic	M	69	R THR 1-stage
28	Aseptic	M	56	L TKR 1-stage
29	Aseptic	F	68	L TKR 1-stage
30	Aseptic	F	76	L THR 1-stage
31	Aseptic	M	64	L THR 1-Stage
35	Aseptic	F	72	R TKA
11	Infected	F	84	L TKR irrigation and debridement with exchange of modular components
14	Infected	F	71	R TKR irrigation and debridement with spacer exchange
15	Infected	M	61	L TKR resection and placement of spacer
17	Infected	F	73	R TKR resection and placement of spacer
18	Infected	M	63	R THR resection and placement of spacer
19	Infected	M	63	L THR resection and placement of spacer
20	Infected	F	61	L THR 1-Stage
21	Infected	M	62	R TKR resection and placement of spacer
23	Infected	M	78	R TKR resection and placement of spacer
32	Infected	M	67	L THR resection and placement of spacer

Summary of the patient characteristics including cohort, age, and procedure received.

*TKA* total knee arthroplasty, *THA* total hip arthroplasty, *TKR* total knee replacement, *THR* total hip replacement, *L* left, *R* right, *B/L* bilateral, *M* male, *F* female.

### Sample collection and preservation

Sample collection for this study was performed after obtaining Institutional Review Board approval. All patients consented to participate in the study. Peripheral blood samples were collected from patients in the preoperative area in Vacutainer Collection tubes with Sodium Heparin (Becton, Dickinson and Company, Franklin Lakes, NJ), with collection volumes ranging from 2 to 4 mL. A volume of 10–20 mL synovial fluid was aspirated from the hip or knee joint and collected in sterile, nuclease-free 50 mL conical copolymer polypropylene screw-cap centrifuge tubes. Upon collection, an equal volume of DNA/RNA Shield (Zymo Research, Irvine, CA) was added to each volume of synovial fluid for sample preservation. Samples were deidentified, stored on ice, and then shipped overnight with ice packs to Contamination Source Identification laboratories (Huntingdon, PA) for sample preparation. Further details regarding synovial fluid, blood, skin swab, and negative control skin and air samples can be found in Supplementary Materials.

### Analytical validation, sample preparation, library preparation, and illumina sequencing

All methods describing analytical validation of metatranscriptome (MT) sequencing using ERCC RNA controls are described in Supplementary Materials. Details regarding sample collection, preservation, preparation for each NGS method, and Illumina sequencing can also be found in Supplementary Materials. For synovial fluid, blood, and negative control saline solution samples, DNA and RNA were extracted for downstream 16S, MG, and MT sequencing. For the negative control skin and air swabs, only DNA was extracted for 16S and MG analysis. For a list of all patient samples processed see Table 1 and for a list of all samples including controls see Supplementary Table S2.

### Bioinformatic analysis

A description of all bioinformatics and statistical analyses for 16S, MG, and MT datasets are described in the Supplementary Materials. Briefly, MT and MG sequence data were subject to quality filtering and adaptor trimming, human sequence removal, and annotation in Kraken2^21^. 16S sequences were merged, de-noised, quality filtered and grouped into ASVs within the DADA2 software package^22^.

Normalization and statistical analyses on 16S data were performed within QIIME2 and R^23^. Microbial annotation count data underwent cumulative sum scaling (CSS) normalization prior to unsupervised and supervised clustering of 16S, MG, and MT data. Statistical differences in clustering between groups were evaluated using the PERMANOVA test. To account for contaminant sequences, CPM normalized ratios of sample:controls counts of matched taxa were calculated prior to random forest modeling. To screen for the presence of antibiotic resistance genes, filtered microbial reads were aligned against the Comprehensive Antibiotic Resistance (CARD) Database via BLAST (min e-value = 1e−10) to detect antibiotic resistance genes in MG and MT data for each sample^24^.

## Results

### Validation and sequencing results

**Figure 1:**
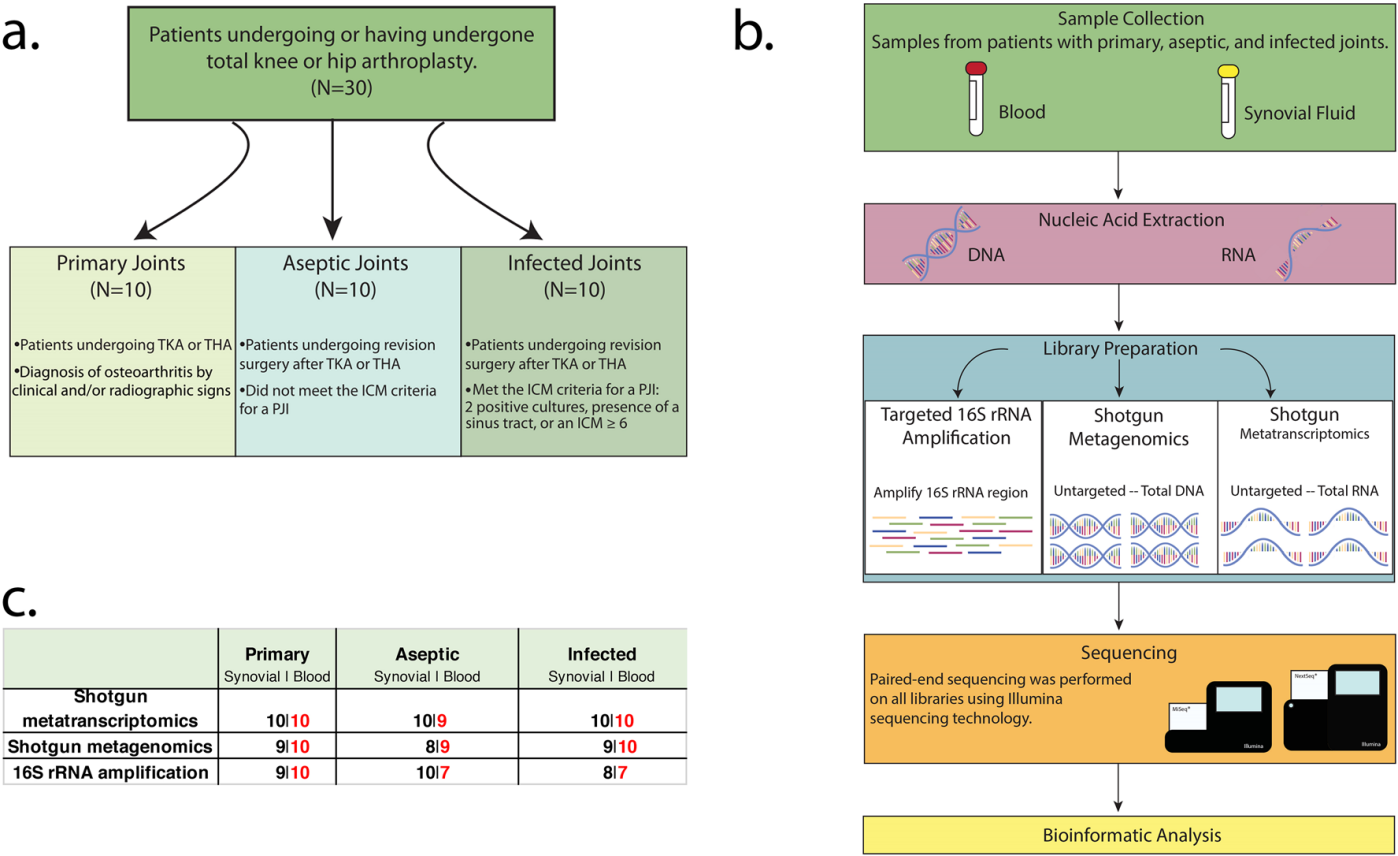
(**a–c**) Study design and workflow. An overview of the study design and workflow. (**a**) Flowchart of the three patient cohorts from which samples were collected and defining characteristics of each cohort. (**b**) The workflow used to profile the microbial communities within synovial fluid and blood samples using three separate library preparation methods: 16S rRNA amplification, shotgun metagenomics, and shotgun metatranscriptomics. (**c**) A breakdown of the number of synovial fluid and blood samples included in downstream bioinformatic processing for each cohort and library preparation method. These diagrams were generated using Adobe Illustrator version 23.0.2 (https://adobe.com/products/illustrator) and Photoshop version 21 (https://www.adobe.com/products/photoshop).

**Figure 2:**
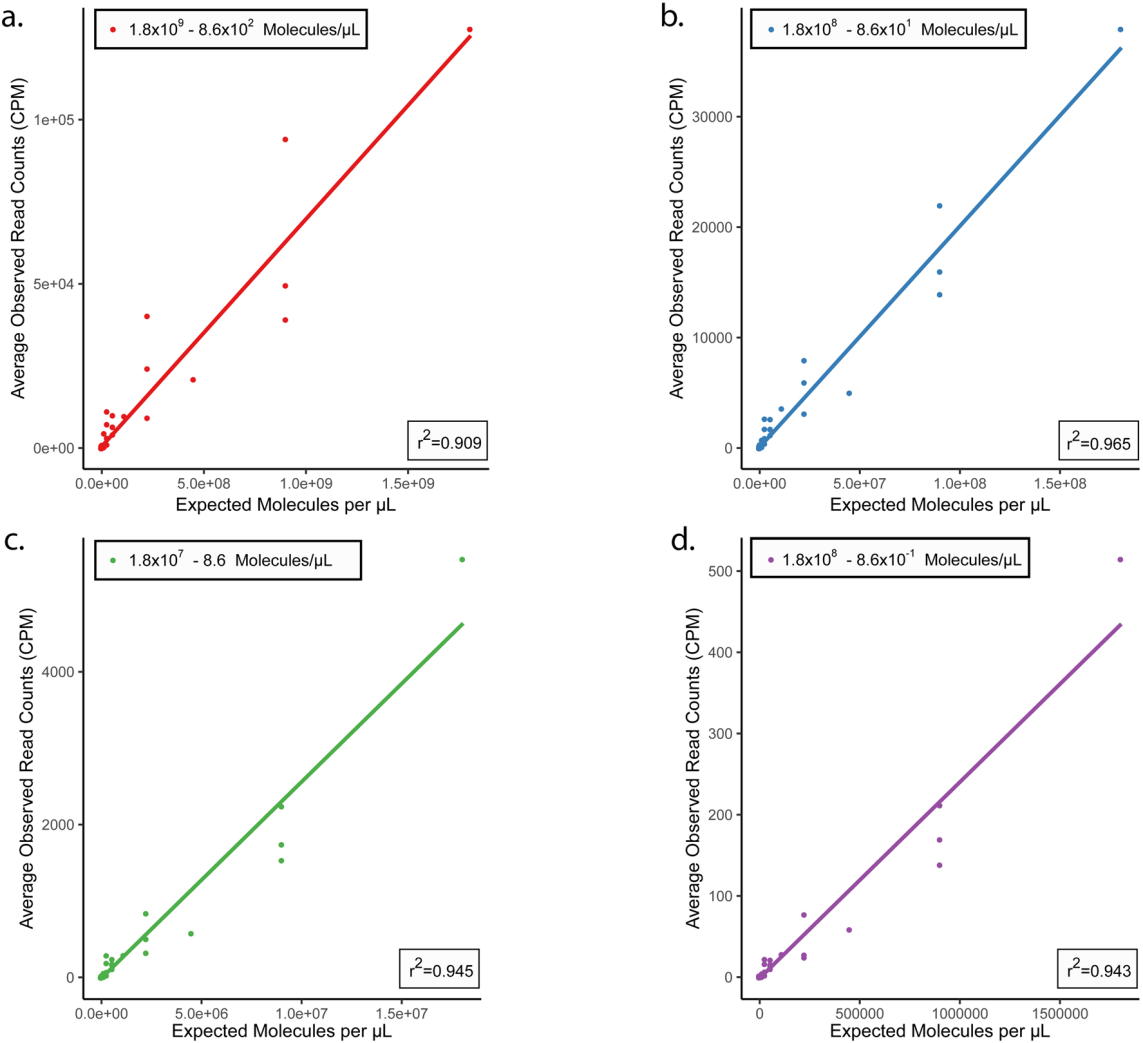
(**a–d**) Validation of metatranscriptomic approach in synovial fluid. Results of the analytical validation experiments using four distinct tenfold serial dilutions of the ERCC RNA spike-in control mix in RNA extracted from synovial fluid. Three replicates were performed at each dilution level with sequences classified as ERCC or non-ERCC using the k-mer based annotation tool CLARK. (**a–d**) The correlation between expected molecules per µL of each ERCC transcript and the average observed read counts of each unique ERCC transcript at concentrations of 1.8 × 10^9^–8.6 × 10^2^ molecules/µL (**a**), 1.8 × 10^8^–8.6 × 10^1^ molecules/µL (**b**), 1.8 × 10^7^–8.6 molecules/µL (**c**), and 1.8 × 10^6^–8.6 × 10^–1^ molecules/µL (**d**).

A total of 30 synovial fluid samples from knee or hip joints and 30 paired blood samples were subjected to 16S rRNA gene amplicon (16S), metagenomics (MG), and metatranscriptomics (MT) library preparation and sequencing (Fig. 1). Due to insufficient DNA or RNA concentrations for library preparation, some synovial fluid or blood samples could not be processed (Fig. 1c). To define the applicability of MT in synovial and whole blood matrices, an analytical validation experiment was performed using ERCC RNA spike-in controls as previously described^25,26^. Validation results showed linear correlations between expected concentration of each ERCC transcript and average observed read counts in synovial fluid (Fig. 2) and whole blood (Supplementary Fig. S1). Strong correlations were found between expected transcripts per µL calculated from positive ERCC controls and observed CPM normalized counts. Within synovial fluid, four distinct tenfold serial dilution experiments yielded linear r^2^ values ranging from 0.909 (1.8 × 10^9^–8.6 × 10^2^ reads/µL) to 0.965 (1.8 × 10^8^–8.6 × 10^1^ reads/µL) (Fig. 2). Within whole blood, linear r^2^ values ranged from 0.886 (1.8 × 10^9^–8.6 × 10^2^ reads/µL) to 0.980 (1.8 × 10^7^–8.6 reads/µL) (Supplementary Fig. S1).

### Beta diversity of synovial and blood microbiota

**Figure 3:**
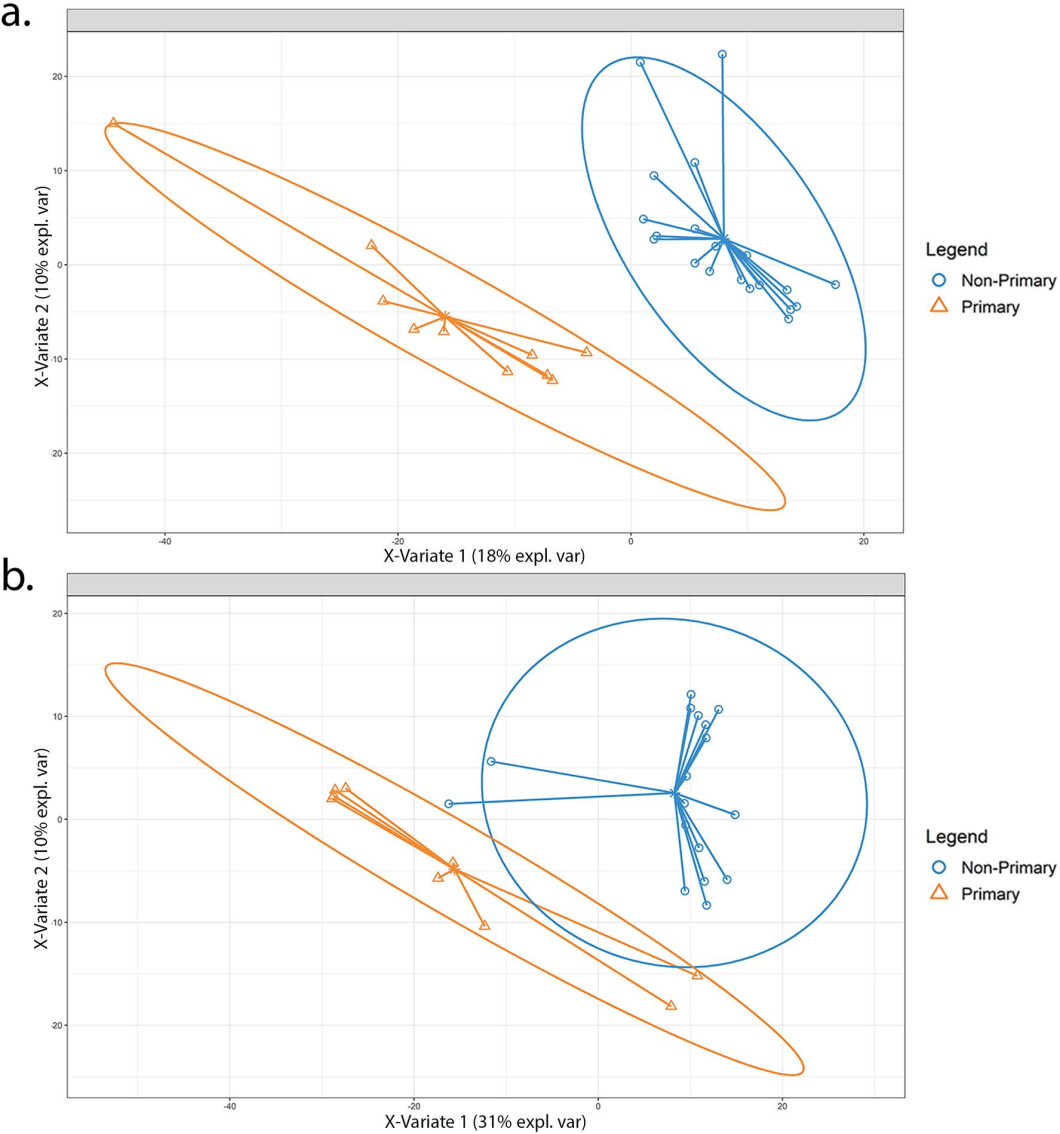
PLS-DA model of primary vs non-primary synovial fluid samples. Partial least squares discriminant analysis (PLS-DA) was conducted within the mixOmics R-package utilizing a CSS normalized counts table of taxon annotations identified using metatranscriptomic [MT] (**a**) and metagenomic [MG] (**b**) synovial fluid samples. The solid ellipses around sample groups indicate 95% confidence. Within the metatranscriptomic dataset we observe separation of primary and non-primary samples while a decrease in separation and differentiation is seen within metagenomic samples.

Unsupervised principal coordinates analysis (PCoA) and supervised partial least squares discriminant analysis (PLS-DA) modeling were conducted to assess differences in microbial communities among primary, aseptic, and infected samples within synovial fluid and matched blood specimens (Fig. 3a,b, Supplementary Figs. S2a–f, S3a,b). Distinct microbial communities exist in synovial fluid MG and MT samples, as supported by significant clustering among primary, aseptic, and infected samples (PERMANOVA, p = 0.001), while a lesser degree of clustering was observed in the 16S dataset (PERMANOVA, p = 0.025). PCoA of blood samples revealed MT had the most significant clustering (PERMANOVA, p = 0.007), followed by MG (PERMANOVA, p = 0.032), and 16S (PERMANOVA, p = 0.049).

Distinction between primary and non-primary samples was observed from MT synovial fluid PLS-DA model (Fig. 3a), as no overlap is observed between 95% confidence ellipses plotted around primary and non-primary clusters (error rate: 11–14%). Increased overlap between primary and non-primary confidence ellipses were noted for MG synovial fluid, resulting in a poorer model performance (error rate: 20–25%) compared to the MT PLS-DA model (Fig. 3b). PLS-DA models built using MT and MG data from blood samples revealed overlap between groups, indicating reduced model performance in differentiating between cohorts compared to models generated using synovial fluid samples.

### Random forest modeling

MT analysis yielded the most accurate predictive random forest models considering both synovial fluid and blood (respectively 79.0% and 75.2%) compared to MG (74.3%, 68.6%) and 16S (38.6%, 51.0%). *Rhizobiales* and *Archromobacter* were among the top predictors for classifying MT blood samples, while *Bacteroidales* and *M.intenstinales* were most predictive for MT synovial fluid samples. A comprehensive list of top predictors of synovial fluid and blood sample groups is in Supplementary Table S3.

### Detection of PJI associated pathogens and synovial fluid culture concordance

**Figure 4:**
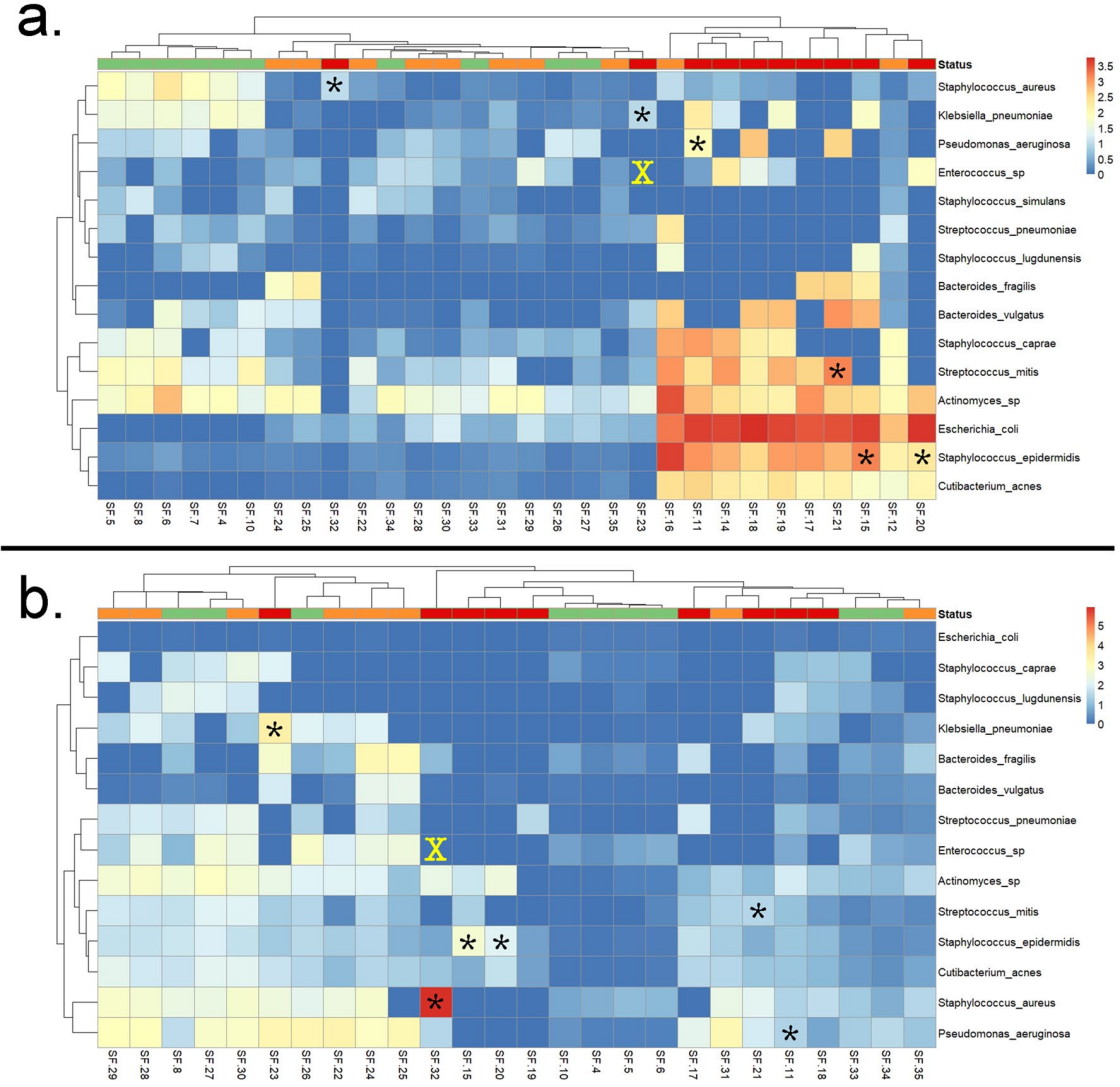
Heatmap of 15 observed clinically relevant PJI-associated pathogens. Samples by group are depicted above the heatmap, with the following colors: orange (aseptic joints), red (infected joints), and green (primary joints). Heatmap of PJI-associated pathogens observed in metatranscriptomic [MT] (**a**) and metagenomic [MG] (**b**) synovial fluid samples. Boxes represent CPM-r normalized counts of each pathogen within synovial fluid samples. Stars (*) represent pathogens identified by library preparation and culture-based methods while an X indicates that the pathogen was identified by culture but not sequencing. Infected synovial fluid samples from the MT dataset yielded differential clustering from remaining samples, with only one infected sample (SF-32), plotting distinctly from other infected samples (**a**). Heatmaps were generated using pheatmap version 1.0.12 (https://cran.r-project.org/web/packages/pheatmap/pheatmap.pdf) and ggplot2 version 3.3.5 (https://cran.r-project.org/web/packages/ggplot2/ggplot2.pdf) within R 3.6.1 (https://www.R-project.org/). Parts of the heatmap marked with stars (*) and X were added using Adobe Photoshop version 21 (https://www.adobe.com/products/photoshop).

Differential clustering of infected MT samples appears to be driven by increased *E. coli* expression (LDA = 4.04, p = 0.00094) in infected samples, compared to primary and aseptic samples (Fig. 4a, Supplementary Table S4). *S. epidermidis* (LDA = 3.55, p = 0.004) exhibited significantly higher abundance within non-primary MT samples compared to primary joints. Of the 7 bacterial taxa identified by culture, 6 were detected using MT sequencing (Fig. 4a, Supplementary Table S4). Sample SF-23 yielded partial concordance with culture, as this sample was culture-positive for *K. pneumoniae,* which was detected, as well as vancomycin resistant *Enterococcus* sp., which was not detected (Supplementary Table S5). However, sequences for the SF-23 MT sample yielded 95% identity with *Enterococcus* but could not be differentiated from alternative genera within the Lactobacillales order. While the MG dataset yielded the same concordance with culture as MT, MG samples clustered indistinctly by cohort. Interestingly, the conserved signature of high abundance of *E. coli* within the infected synovial fluid samples of the MT dataset was not observed within the MG SF data (Fig. 4b, Supplementary Fig. S4). Notably, this was likely due to true biological signal and not contamination, as 2000 of the 2032 contigs (assembled using only sequences initially identified as *E. coli*) were classified by BLAST (with NCBI’s nucleotide database) once more as *E. coli.*

### Antibiotic resistance gene screening of synovial fluid

**Figure 5:**
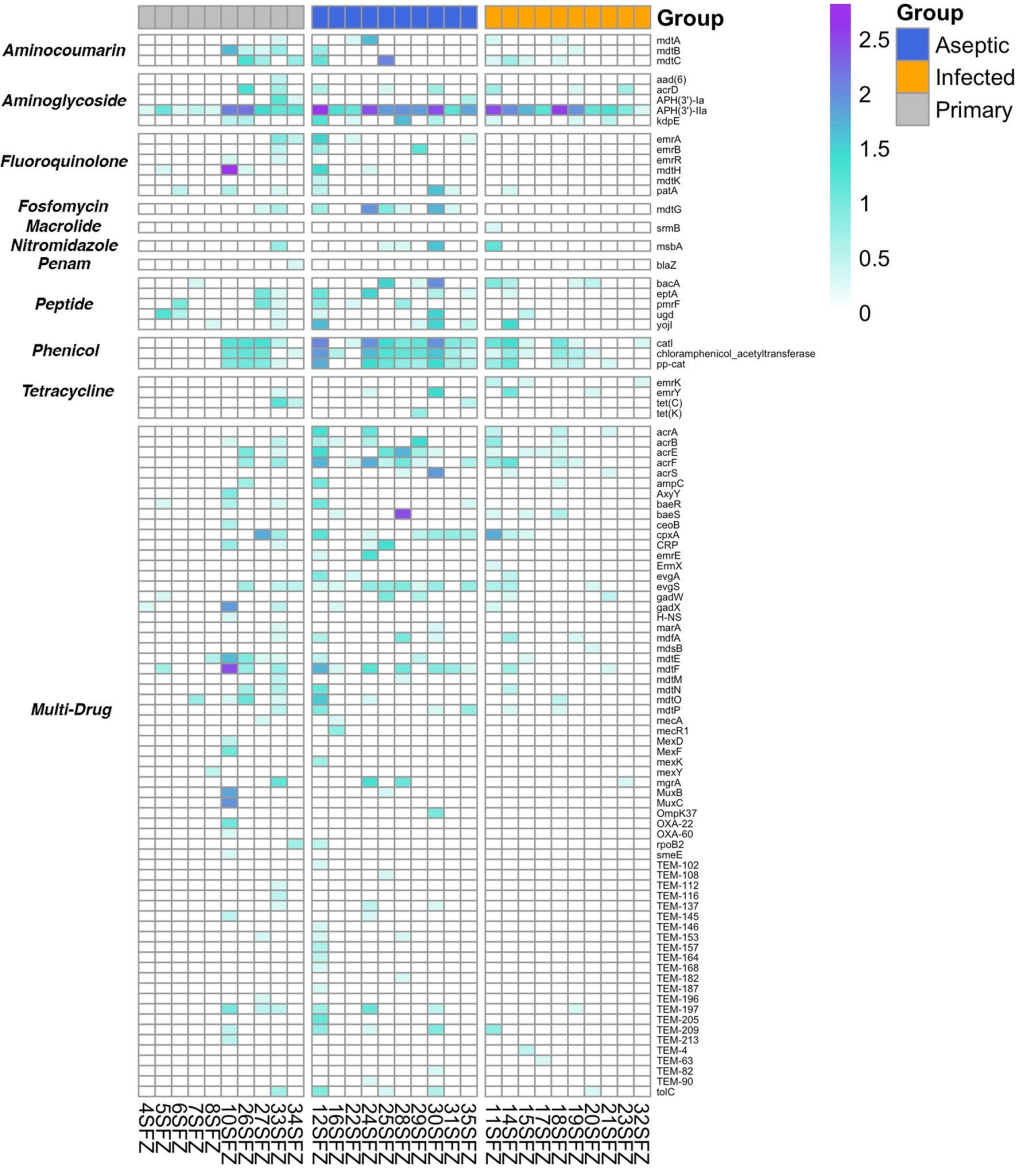
Antibiotic resistance gene (ARG) heatmap. Heatmap displaying log transformed counts of antibiotic resistance genes identified to be actively expressed within each synovial fluid sample when considering the MT dataset. The y axis displays the observed ABX resistance gene, which are stratified by the respective antibiotic to which resistance is conferred. Samples are stratified along the X-axis by their respective “status” and are ordered from left to right as *primary joints, aseptic revisions,* and *infected revisions,* respectively. This heatmap was generated using pheatmap version 1.0.12 (https://cran.r-project.org/web/packages/pheatmap/pheatmap.pdf) and ggplot2 version 3.3.5 (https://cran.r-project.org/web/packages/ggplot2/ggplot2.pdf) within R 3.6.1 (https://www.R-project.org/).

MT SF yielded 85 ARGs across all samples (Fig. 5), while culture yielded positive results within 4/10 infected samples (samples 15, 20, 21, 23). Among infected samples, concordance between MT and culture was observed, whereas MG yielded no concordance to culture. Patient 15 culture results revealed Oxacillin-resistant *Staphylococcus epidermidis*, and similarly in the MT dataset, penam class antibiotic resistance genes *acrE* and *mdtE* (Oxacillin resistance) were identified. Cultures from Patient 20 were positive for tetracycline and erythromycin resistance. While the MT profile yielded expression of both tetracycline class (*evgS, tolC, emrY*) and erythromycin class (*evgS*, *tolC*) ARGs for patient 20. Similarly, patient 21 concordance of erythromycin resistance was observed for MT and culture, in addition to two macrolide class genes (*mdtF*, *gadW)*. MG profiles of patients 15, 20, and 21 failed to identify ARGs. Vancomycin resistance gene expression was not observed within the MG and MT data from patient 23, and therefore neither MG nor MT analysis were concordant with culture results. Two aminoglycoside class ABX resistance genes (*APH[3i]-Iia*, *acrD*) and one multidrug resistance gene (*mgrA*) were annotated from MT microbial reads obtained from patient 23.

### Assessment and removal of environmental contamination

**Figure 6:**
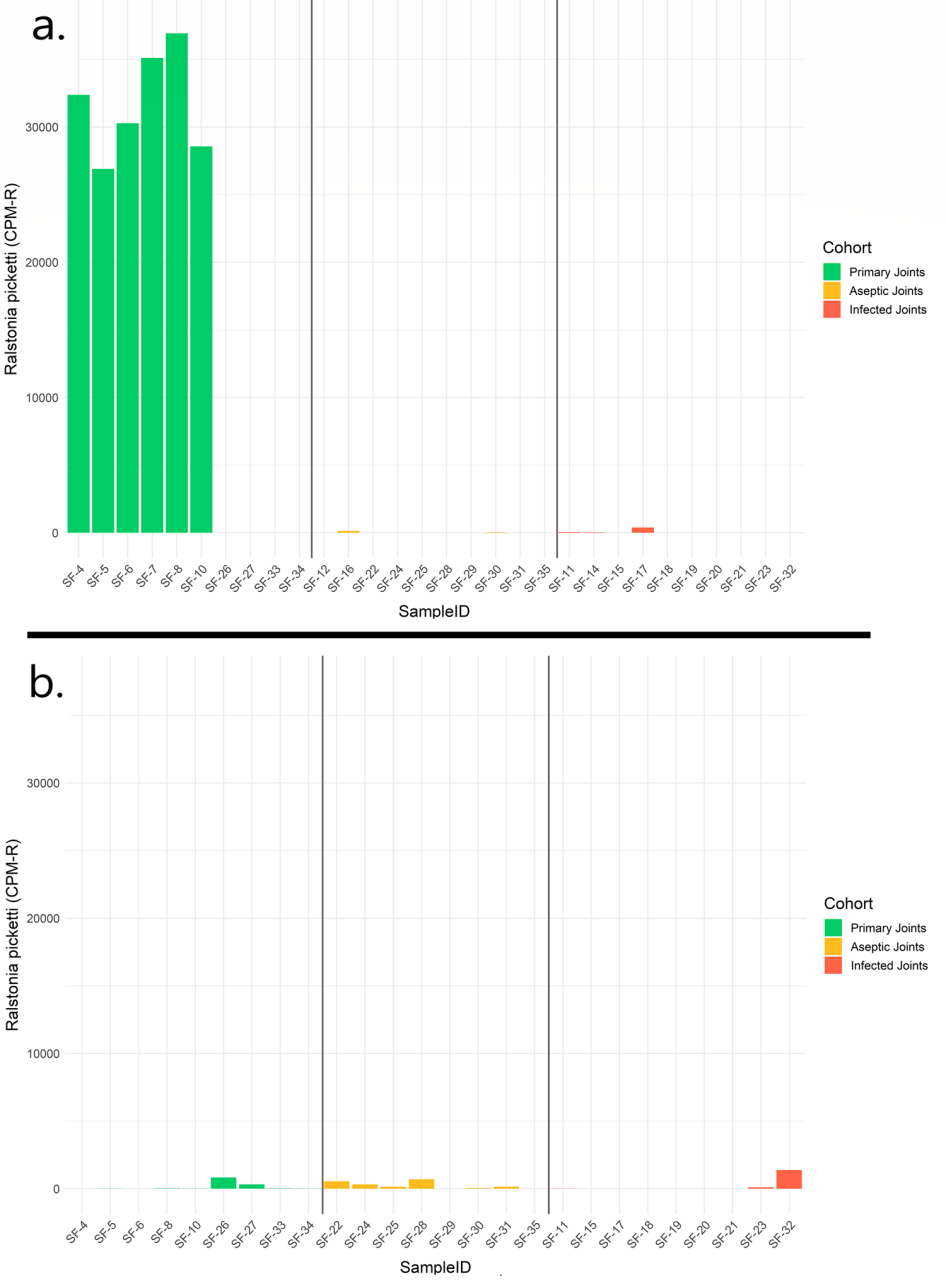
*Ralstonia picketti* relative abundance plots. Plots showing the CPM-r normalized abundance of *Ralstonia picketti* in metatranscriptomic [MT] (**a**) and metagenomic [MG], (**b**) synovial fluid samples. Primary MT samples had the highest abundance of *Ralstonia picketti*, with the species present in low abundance or completely absent in all other MT or MG cohorts.

Due to the high potential of environmental contamination within untargeted microbial genomic investigations, rigorous environmental controls were collected to assess the microbiome of the respective environments encountered during the sample collection process. Considering the MG dataset, consistent contaminating microorganisms of prominent (top 10) abundance were observed across sampled skin swabs, air swabs, and NaCl (Supplementary Fig. S5). An unclassified taxon within the Clostridiales were identified as the most prominent contaminant within both the air swab and NaCl samples (average relative abundance of 10% and 14%, respectively), whereas the *Cutibacterium acnes* were the prominent contaminant within the skin swab MG samples (average relative abundance of 19%). The *Cutibacterium acnes* were also observed as a prominent contaminant when considering the MT dataset, and comprised 13%, 9%, and 14% community abundance within skin swab, NaCl, and air swab samples, respectively. The fungi *Malassezia restrictica*, which was not observed as a prominent contaminant within the MG dataset, was identified at 9%, 5%, and 10% abundance within skin swab, NaCl, and air swab samples, respectively within the MT data. Blank samples were included on each sequencing run to account for potential contamination biases and to control for environmental organisms. After CPM-r normalizing our data with run-specific blank sample annotation data, a consistent signature of *Ralstonia picketti* was discovered within the primary joints of the MT dataset (Fig. 6). A total of 6 out of 10 primary joints yielded a consistent profile of elevated *Ralstonia picketti* abundance in comparison to the remaining samples. The trend of increased *Ralstonia* abundance was not observed within the MG samples, nor identified as a prominent environmental contaminant within either the MG or MT datasets. Consequently, this taxon was identified as the most significantly enriched species within the primary joints when considering the MT dataset (LDA score = 3.90, p = 0.008).

## Discussion

PJIs are a complication for patients undergoing total hip (THA) and knee (TKA) arthroplasty and continue to pose a public health concern since current methods lack accuracy to identify causative pathogens. Pitfalls associated with current assays mandate a robust clinical test system that rapidly and precisely detects pathogens associated with infection. Our study assessed: (1) utility of NGS technologies, including, 16S rRNA gene (16S), metagenomics (MG), and metatranscriptomics (MT) to characterize microbial communities of synovial fluid and blood samples from patients classified under primary, aseptic revision, and revision for PJI, and (2) concordance of NGS to culture-based assays in identifying pathogens and ARGs from clinical specimens. Herein, we described the potential clinical utility of an analytically-validated MT method for identification of active microbial consortia in synovial fluid and blood specimens.

In this study, MG and MT techniques outperformed 16S in distinguishing between sample groups (Supplementary Fig. S2). Moreover, our results revealed MT as a superior technique, yielding the most accurate random forest (RF) models and differentiation between primary and non-primary groups. Top predictive taxa for classifying MT samples using our RF model, revealed several taxa associated with surgical infections^27,28^. These findings highlight how MT-based classification models can simultaneously identify several active biomarker taxa associated with PJIs. Unlike MG and 16S, MT captures the active microbial profile by sequencing and annotating RNAs from a specimen, circumventing issues associated with DNA-based methodologies^29^. Our results suggest that identifying active organisms can be a promising discriminator and relevant clinical tool, particularly with PJI where treatment can be focused on highly active microorganisms.

MT profiling of clinical samples revealed superior taxonomic resolution of microbial consortia compared to MG and 16S methods. Moreover, MT exhibited greater differential quantitative signals (i.e., relative abundance) compared to the other methods. Since MG generates sequence data from all DNA present in a sample, this method is prone to false positives for pathogen detection^29,30^. These false positives occur because DNA degrades over time, and inactive microbial populations, or naked DNA, remain detectable using MG. The greater discrimination from MT is likely because it generates sequence data from active transcripts, thus avoiding potentially latent, dead, or inactive microbial populations^29^. For instance, infected joints subject to MG sequencing exhibited an increased relative abundance of several PJI-associated taxa^31–33^. While *S. epidermidis* and *C. acnes* are both skin commensals, they are also associated with PJIs due to the invasiveness of prosthetic joint procedures. Notably, these taxa can exhibit elevated ARGs from patients with PJIs compared to similar strains on the skin of healthy patients^34^.

Although no infected samples returned culture positive for *E. coli*, biomarker enrichment analysis of MT data revealed *E. coli* to be significantly enriched in infected samples and least expressive in primary joints. Infected samples may not have been able to return culture positive for *E. coli* due to sensitivity limitations associated with culture-based methods^35^. The possibility of cross-contamination is unlikely since the signal of *E. coli* in negative controls were minimal. One case study isolated variants of *E. coli* from a PJI patient with history of recurring UTIs involving *E. coli* and *K. pneumoniae*, implying UTIs could be a contributing factor for increased expression of *E. coli* among infected joints^36^. Contrary to current literature, our results imply that other factors may be contributing to *E. coli* expression in infected joints from this study, since there were no reports of UTIs among our cohort. Apart from known risk factors like UTIs and surgical area proximity to gastrointestinal tract, MT methods can serve a crucial role in addressing uncertainties surrounding identification of risk factors and treatment of *E. coli*-associated cases of PJI.

Additionally, our study revealed a potential underlying “native” joint microbial community. MT data showed highest expression of *Ralstonia* spp. in primary joint specimens. Although the genus *Ralstonia* has been identified as a contaminant in other studies^37,38^, *Ralstonia picketti* was not observed to be among the top 10 most abundant taxa in any of our controls (Supplementary Fig. S5), in contrast to its relatively high CPM-r abundance in the primary joints subject to MT (Fig. 6). Additionally, *S. aureus* and *K. pneumoniae* were also more abundant in primary joints, relative to non-primary joints. Native joint space was historically thought to be a sterile environment, void of any microorganisms, but recent literature has unveiled the existence of an endemic microbiome^39,40^. A study of 40 patients undergoing primary TKA, 12 were found to have at least one organism identified using NGS^40^. Results of our study support these findings and suggests that the joint microbiome differs based on the presence of a prosthesis.

Results from this study revealed that MT yielded a high culture-positive concordance of 83%. The single case of partial concordance in our study could be due to multiple reasons, including underdeveloped databases, quality of sample, and inconsistencies with culture-based assays^41^. Of particular note, the microbe not identified by MT, a vancomycin resistant *Enterococcus* sp., was isolated via culture from broth only. Previous reviews of test characteristics have demonstrated the poor clinical utility and reliability of broth-only isolates which draws into question the reliability of the identification of this non-concordant microorganism^42^. Additionally, MT was also superior in ARG detection and concordance with culture antibiotic susceptibility testing, as it detected ARGs in 3 cases concordantly with culture where MG failed to identify ARGs overall. In particular, Aminoglycoside resistance genes were expressed across all groups, whereas Phenicol resistance was largely observed in aseptic joints. Interestingly, bBoth drug classes contain wide-spectrum antibiotics that have been used in treatment of PJIs and infections relating to eyes and urinary tract^43–45^.

Regarding ARG, a broad range of resistance mechanisms were noted throughout the study. In particular, Aminoglycoside resistance genes were expressed across all groups, whereas Phenicol resistance was largely observed in aseptic joints. Interestingly, both drug classes contain wide-spectrum antibiotics with Aminoglycosides being used in treatment of PJIs and Phenicols being utilized for infections relating to eyes and urinary tract^43–45^. This finding can possibly be explained due to the absence of selective pressures on the different cohort’s microbiome. Patients within the aseptic joint cohort were not previously treated with any courses of antibiotics or operative procedures with the goal of treating PJI. Phenicol resistance could have mainly been observed among aseptic joints due to the lack of selective pressure of a more common broad spectrum antibiotic. Several of the ARG genes noted in this group are protective against older and less commonly used antibiotics (i.e., chloramphenicol) therefore their expression may be found more commonly in the aseptic cohort when compared to the septic revision cohort. Additionally, there were some multidrug resistant mechanisms noted in this aseptic cohort. This study utilized the ICM criteria to define the presence or absence of PJI, however as newer diagnostic techniques emerge it is possible that some of the aseptic cases may have indeed been due to an underlying infection. With the advent of newer, more sensitive techniques, such as MT, and with further clinical research a future update to the diagnosis of PJI may rely more heavily on laboratory diagnostic data. This is of course out of scope of this study and further work with larger cohorts and longer clinical follow up are needed.

Within the scope of this study, our results suggest MT can provide the unique ability to accurately detect microbes and ARGs to a greater extent than other existing techniques. As is the case in all infectious disease, accurate identification of resistant strains is crucial to ensure appropriate antibiotic treatment is administered.

While these techniques can detect low levels of microbes, it is important to reduce the risk of sample contamination. Analysis of negative controls, air, and skin swabs demonstrated that contaminants are relatively common. Of the prominent contaminants identified, *C. acnes* is a known common PJI-associated pathogen, particularly in total shoulder arthroplasty, although to a much lesser extent than other common pathogens, *S. aureus* and *S. epidermidis*^33,46,47^. False positive results involving common pathogens can lead to incorrect PJI diagnoses, which may incur unnecessary procedures and treatment for patients. As demonstrated in this study, using blank samples enable controlled analyses by revealing contaminant organisms, thus reducing the effect of contamination on final results.

This study is not without limitations. Since 16S sequencing requires selecting primers targeting hypervariable regions of the gene, primers for different regions than the ones used in this study may yield different results. Within this study, blood and synovial fluid were collected, but it is unknown if swabbing of infected tissue/implants would impact results. Though other studies have depleted human DNA from clinical samples before extraction to improve microbial signal yield^48^, a human DNA depletion step did not occur during this study. Considering these limitations, we demonstrate the potential for MT to offer significant improvements over 16S sequencing and even MG in the detection of active microorganisms and ARGs. Future clinical validation studies are warranted to establish the utility of MT as a clinical diagnostic test. Preliminarily, this study suggests MT may be a valuable method for diagnosing suspected PJIs and able to improve detection of pathogens and ARG identification. Future work towards the development of a clinically valuable assay should investigate the efficacy of MT for diagnosing clinical disease states, including PJIs. Analytical validation experiments would be required to assess the limit of detection, precision, sensitivity, and specificity of this technology when applied to blood and synovial samples with known outcomes, similar to the analysis done in other NGS assay validation experiment^1,4^. Additionally, a clinical validation trial would be conducted to assess the assay’s diagnostic capabilities with real patient samples once the analytical thresholds of the assay have been derived.

## Supplementary Information

Supplementary Information 1.

Supplementary Information 2.

Supplementary Information 3.

Supplementary Information 4.

Supplementary Information 5.

Supplementary Information 6.

## Data Availability

All raw files are available at NCBI’s Short Read Archive under accession number SUB9220509.
