# Supplementary material for: Comparative meta-omics for identifying pathogens associated with prosthetic joint infection
Source: Sci Rep. 2021 Dec 9;11:23749. doi: 10.1038/s41598-021-02505-7 (PMC8660779; doi:10.1038/s41598-021-02505-7)
Supplement: Supplementary file 1 — Supplementary Information 1. [file 41598_2021_2505_MOESM1_ESM.docx]

**Supplementary Information**

Comparative Meta-omics for Identifying Pathogens Associated with Prosthetic Joint Infection

Karan Goswami MD^1#^, Alexander J. Shope MD^1,2#^, Vasily Tokarev^2#^, Justin R. Wright^2#^, Lavinia V. Unverdorben^2^, Truc Ly^2^, Jeremy Chen See^2^, Christopher J. McLimans^2^, Hoi Tong Wong^2^, Lauren Lock^2^, Samuel Clarkson MD^1^, Javad Parvizi MD^1*^, Regina Lamendella^2,3*^

^1^Rothman Institute, Philadelphia, PA, USA

^2^Contamination Source Identification LLC, Huntingdon, PA, USA

^3^Juniata College, Huntingdon, PA, USA

# Karan Goswami, Alexander J. Shope, Vasily Tokarev, and Justin R. Wright are co-first authors and contributed equally to this article. Order was determined alphabetically by last name.

*Address correspondence to:

Dr. Regina Lamendella, [reginalamendella@csidx.com](mailto:reginalamendella@csidx.com)

Dr. Javad Parvizi, [javadparvizi@gmail.com](mailto:javadparvizi@gmail.com)

**Supplementary Tables**

Supplementary Table S1: ICM Classification Data. Summary table of all applicable physical exam and laboratory findings for ICM classification of PJI-Aseptic and Septic cohorts.

Supplementary Table S2: Sequencing Results for 16S rRNA gene amplicon (16S), metagenomics (MG), and metatranscriptomics (MT). The 16S, MG, and MT sequencing results for all processed patient samples and negative controls. MT sequencing yielded the highest number of raw reads and annotated microbial reads compared to MG and 16S.

Supplementary Table S3: Top Random Forest Predictors for Synovial Fluid and blood samples. Table of the top predictors for classifying metatranscriptomic synovial fluid and blood samples, with most predictive bacterial taxa at the top of the table and least predictive at the bottom.

Supplementary Table S4: Linear discriminant analysis Effect Size (LEfSe) results for Synovial Fluid and Blood Samples across all conducted genomics analyses (16S rRNA gene amplicon, metagenomics, and metatranscriptomics).

Supplementary Table S5: Synovial Fluid Culture Results. The synovial fluid culture results of patients with infected joints, with (-) indicating no bacteria or antibiotic resistance were cultured or identified. Out of ten patient samples, four were culture negative and six samples were culture positive, with five out of the six culture positive samples showing antibiotic resistance. Counts of unique AMR genes for each synovial fluid sample from MT sequencing results.

**Supplementary Figures**


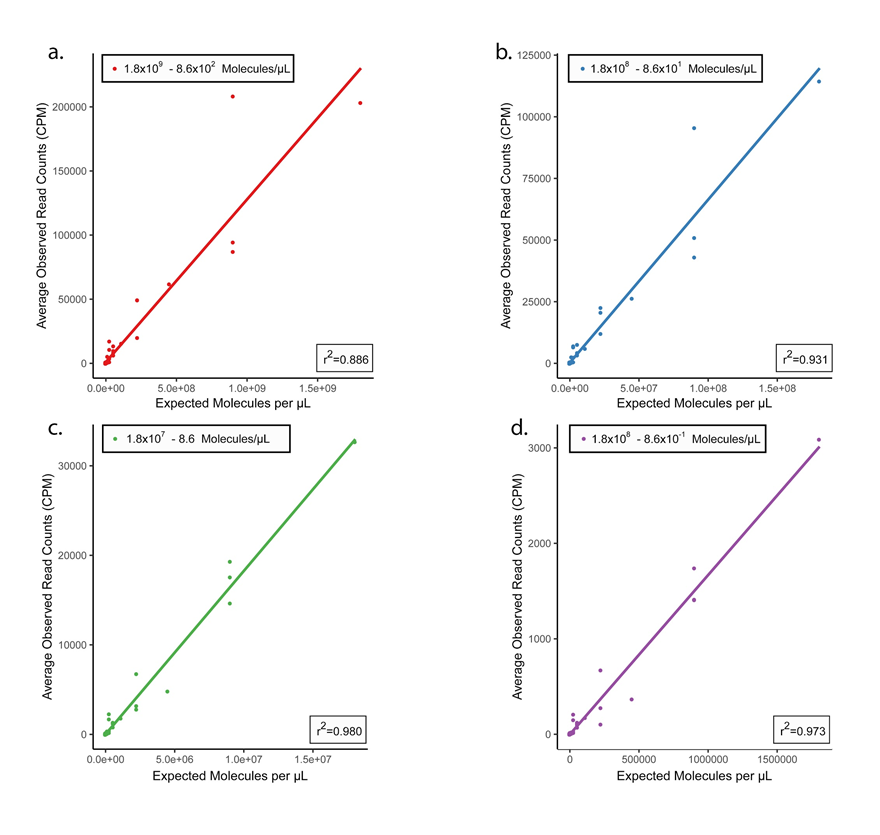


Supplemental Figure S1: Validation of metatranscriptomic approach in whole blood. Results of the analytical validation experiments using four, 10-fold serial dilutions of the ERCC RNA spike-in control mix in RNA extracted from whole blood. Three replicates were performed at each dilution level with sequences classified as ERCC or non-ERCC using the k-mer based tool CLARK. (a-d) The correlation between expected molecules per µL of each ERCC transcript and the average observed read counts of each unique ERCC transcript at concentrations of 1.8x10^9^-8.6x10^2^ molecules/µL, 1.8x10^8^-8.6x10^1^ molecules/µL, 1.8x10^7^-8.6 molecules/µL, and 1.8x10^6^-8.6x10^-1^ molecules/µL.


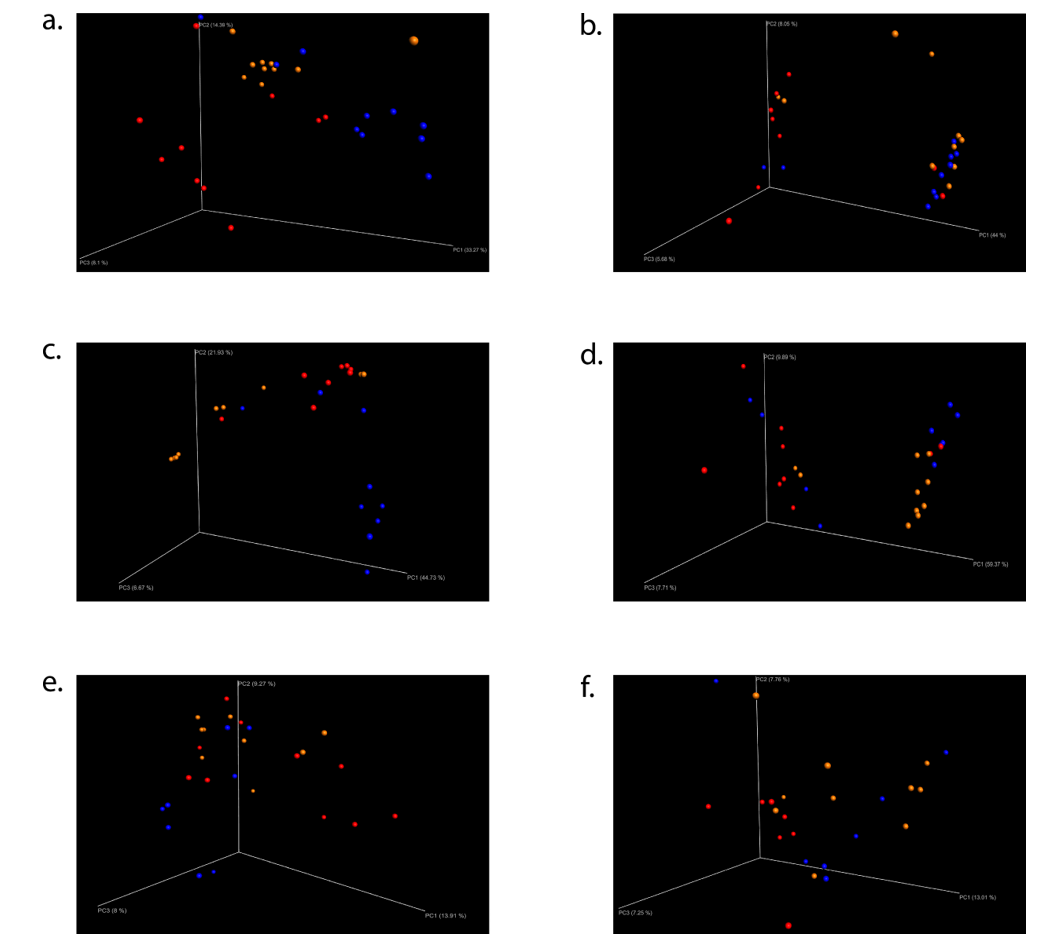


Supplemental Figure S2: Principal coordinate analysis (PCoA) plots of MT, MG, and 16S data. Bray curtis principal coordinate analysis (PCoA) plots showing the differences in the bacterial communities between primary (blue), aseptic (red), and infected joints (orange), with each dot representing a single blood or synovial fluid sample. The density of each cohort on the plot is displayed along the respective axis. Significant differential clustering between the three cohorts is observed for metatranscriptomic [MT], metagenomic [MG], and 16S synovial fluid (a, c, e) and blood (b, d, f) samples. Notably, PC1 axis explained 33.27% of the total variation for MT (a) and 44.73% of variance for MG (c). Blood samples showed that the PC1 axis accounted for 33.27% and 59.47% of variation between cohorts for MT (b) and MG (d), respectively.


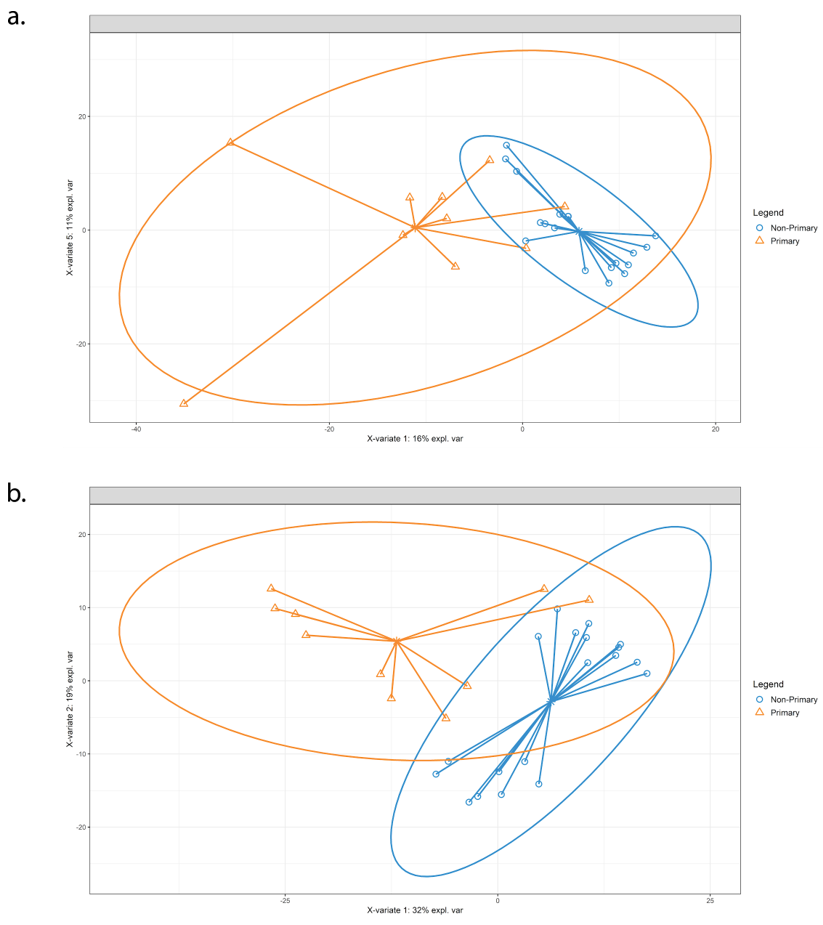


Supplemental Figure S3: PLS-DA plots of primary and non-primary blood samples. Partial least squares discriminant analysis (PLS-DA) was conducted within the mixOmics R-package utilizing a CSS normalized counts table of taxon annotations identified using metatranscriptomic [MT] (a) and metagenomic [MG] (b) blood samples. The solid ellipses around sample groups indicate 95% confidence. We observed overlap of primary and non-primary samples in models using both MT and MG, indicating that these models were unable to differentiate well between the two groups.


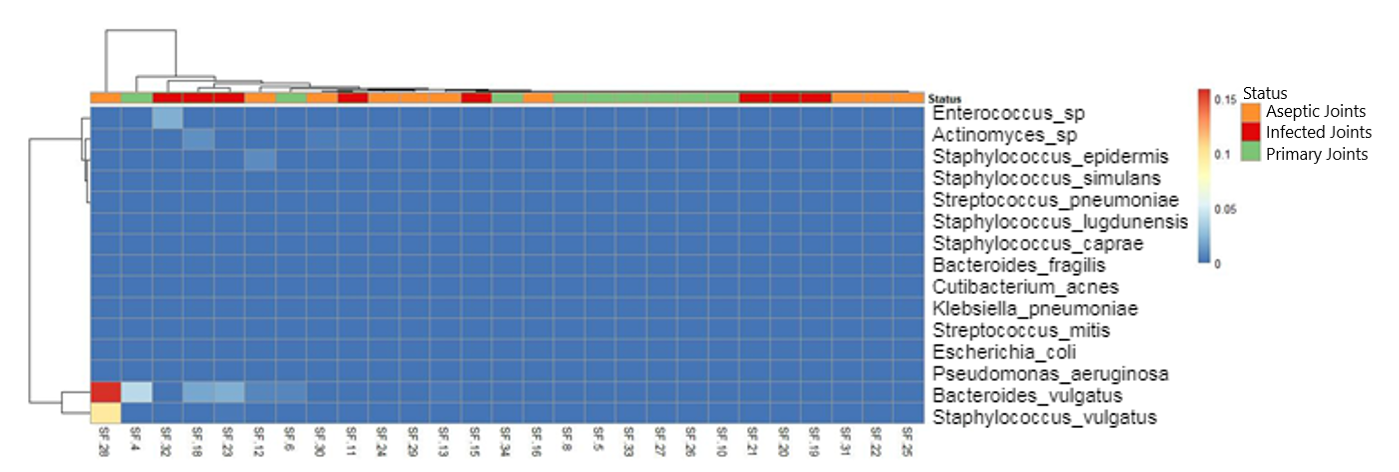


Supplemental Figure S4: Heatmap of observed clinically relevant PJI-associated pathogens in 16S. Heatmap of 15 clinically relevant, PJI-associated pathogens observed in 16S synovial fluid samples. Each box represents the CPM-r normalized counts of each pathogen present within a respective synovial fluid sample. The 16S dataset yielded 0% concordance with synovial fluid culture results. This heatmap was generated using pheatmap version 1.0.12 (https://cran.r-project.org/web/packages/pheatmap/pheatmap.pdf) and ggplot2 version 3.3.5 (https://ggplot2.tidyverse.org) within R 3.6.1 (<https://www.R-project.org/>).


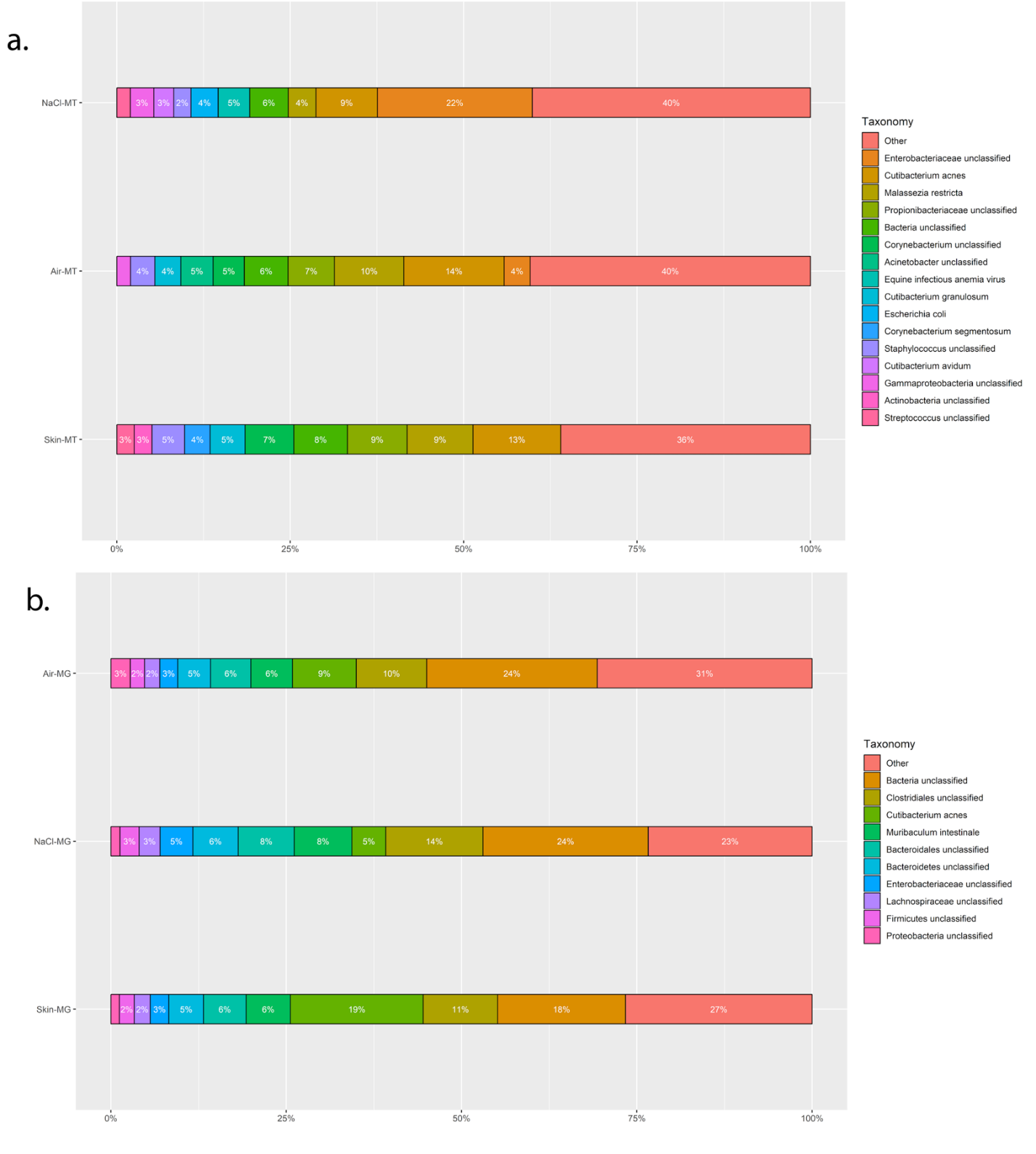


Supplemental Figure S5: Top 10 most abundant contaminating taxa in negative control samples.

The average relative abundance of the top 10 most abundant bacterial taxa in negative control skin swabs, air swabs, and NaCl for metatranscriptomics [MT] (a) and metagenomics [MG] (b). Within the MT samples *Cutibacterium acnes* was a prominent contaminant while an unclassified Clostridales taxon was identified as most prominent in MG samples.

**Supplementary Methods**

ERCC Limit of detection (LoD) for RNA sequencing libraries

The stock ERCC RNA Spike-in Mix (ThermoFisher Scientific, Waltham, MA) contained a mixture of 92 synthetic transcripts with concentrations ranging from 8.6x10^3^ to 1.8x10^10^ transcripts/uL. Using the stock ERCC mixture four 10-fold serial dilutions, (1:10, 1:100, 1:1,000, and 1:10,000) were made using DNase/RNase free water. RNA was extracted from whole blood and synovial fluid and concentrations were quantified using the Invitrogen Qubit 4 Fluorometer and Qubit RNA HS Assay kit (ThermoFisher Scientific, Waltham, MA).

For each sample matrix, 15 RNA extracts (with a concentration of 10 ng of RNA) and two controls (one positive and one negative) were library prepared for sequencing using the Trio RNA-Seq kit (Tecan Genomics, Redwood City, CA). At the beginning of library preparation, of the 15 samples, 12 received a 1uL ERCC spike in, with three replicates per dilution. The three remaining samples did not receive an ERCC spike-in. The protocol for the Trio RNA-Seq kit (Tecan Genomics, Redwood City, CA) was followed as stated. All samples were pooled and sequenced using an Illumina NextSeq instrument with paired-end 150 bp reads.

Synovial Fluid Collection

Synovial fluid samples were all obtained intraoperatively. After the initial superficial dissection, a sterile syringe was used to aspirate the operative joint prior to the arthrotomy. The resulting synovial specimen was then placed into a sterile container and handed off the sterile field. Each specimen was then appropriately marked with a numerical value to blind the lab to the identity of specimen's cohort. Using sterile technique, approximately three milliliters of DNA/RNA Shield (Zymo, California) was added to the specimen container to preserve the DNA and RNA. All specimens were then kept on ice prior to and during shipping.

Blood Collection

Blood samples were collected in the pre-operative area. Prior to collection of the specimen, the tops of the blood tubes were sanitized with alcohol swabs and then allowed to air dry before sample collection. Three milliliters of each patient’s blood were collected into a sterile syringe and then ejected into a vacuum sealed tube containing sodium heparin to prevent coagulation. Each specimen was appropriately labeled with the same numerical identifier as all other specimens associated with each respective patient. Blood specimens were stored on ice prior to shipping and were then shipped to CSI® on ice with their associated synovial fluid and negative control samples.

Skin Swab Collection

After anesthesia was administered and the patient was positioned on the operative table, the skin of the operative limb was prepped with povidone iodine and alcohol. Following standard skin prep, the surgical field was draped in a sterile manner. A skin swab of the surgical site was collected once all drapes were in place by the surgeon by placing the tip of the swab on the area of the skin that was to be incised. The surgeon used a dragging and twisting motion to assure an appropriate sample of the skin flora was collected. The swab was then placed back into its sterile tube and handed off the sterile field. Swab tubes were labeled in the same manner as previously described and then shipped on ice to the lab for analysis.

Air Swab collection

During the surgical procedure, after the arthrotomy of the joint was performed, a researcher outside of the sterile field collected an air sample by removing a swab from its container and holding it upright inside the laminar flow hood surrounding the operative field. It was held stationary in the air for 20 seconds before being placed back in its sleeve and appropriately marked. Care was taken to not allow the swab to come into contact with any surfaces.

Sterile Saline Sample Collection

After negative controls and samples had been collected, a flush syringe of sterile saline was obtained in the operating room. A sealed vacuum container with sodium heparin- the same as used for sampling patient blood- was prepared in the same manner as above. A sterile needle was attached to the tip of the saline syringe and approximately 2-3 mL saline from the flush syringe was placed in the vacuum container. The syringe and needle were properly disposed of and the negative control appropriately labeled.

Sample preparation for 16S rRNA and shotgun metagenomic sequencing

Before every DNA extraction, all work areas were thoroughly cleaned with solutions of 10% bleach followed by 70% ethanol. DNA extraction and isolation of blood samples was performed using the Qiagen DNeasy Blood and Tissue kit (Qiagen, Valencia, CA), according to the manufacturer’s protocol. For each sample between 0.5-1.0mL of blood was extracted. DNA extraction and isolation of synovial fluid, skin and air swabs, and saline solution samples was performed using the Qiagen DNeasy Powersoil kit (Qiagen, Valencia, CA) and the manufacturer's protocol was followed. A volume of 1.0-1.5 mL of synovial fluid was extracted using a single 0.22 µm pore polyethersulfone filter (MilliporeSigma, Burlington, MA) as input, to improve recovery, with that volume being pushed through the sterile filter using a sterile 60 mL syringe (Becton, Dickinson and Company, Franklin Lakes, NJ). For saline solution samples a volume of 0.5-1.0 mL was extracted through a single filter. Samples were eluted using 50 μL of DNase/RNase-free water and DNA concentrations were quantified using the Invitrogen Qubit 4 Fluorometer (ThermoFisher Scientific, Waltham, MA) and Qubit dsDNA HS Assay kit (ThermoFisher Scientific, Waltham, MA).

Sample preparation for shotgun metatranscriptomic sequencing

All RNA extraction was performed in a laminar flow hood with all work surfaces being wiped down first with a solution of 70% ethanol followed by RNaseZap (ThermoFisher Scientific, Waltham, MA) before extraction. RNA from blood, synovial fluid, and saline samples was extracted using the RNeasy PowerMicrobiome kit (Qiagen, Valencia, CA). For blood and synovial fluid samples, a volume of 1.0-1.5 mL was extracted through a single filter to improve recovery. For saline solution samples, a volume of 0.5-1.0 mL was extracted through a single filter. The protocol was followed as stated and samples were eluted with 50 μL of DNase/RNase-free water. RNA concentrations were quantified using the Invitrogen Qubit 4 Fluorometer (ThermoFisher Scientific, Waltham, MA) and Qubit RNA HS Assay kit (ThermoFisher Scientific, Waltham, MA).

Illumina Sequencing

All samples were prepared for 16S rRNA, shotgun MG, and MT sequencing. Library preparation for 16S rRNA sequencing used PCR and was performed as described by Schieffer et al^1^. MG libraries were prepared with the extracted DNA from all sample types and Ovation Ultralow V2 DNA-Seq kit (Tecan Genomics, Redwood City, CA). MT libraries for all samples were prepared using the extracted RNA and Trio RNA-Seq kit (Tecan Genomics, Redwood City, CA), with RNA extracts first being DNase treated per the Trio RNA-Seq kit’s protocol (Tecan Genomics, Redwood City, CA). rRNA depletion was not performed on the extracts prior to library preparation. Libraries were quality checked using the Agilent 2100 BioAnalyzer and the Agilent DNA High Sensitivity DNA kit (Agilent Technologies, Santa Clara, CA). The libraries were then pooled, and gel purified on a 2% gel using the Qiagen Gel Purification kit (Qiagen, Frederick, MD) to size select libraries to a range of 250-400 bp. After purification and dilution to the final concentration, 16S rRNA runs received a 20-25% spike-in of the PhiX V3 control library while MG and MT runs received a 1% spike-in (Illumina, San Diego, CA). All 16S rRNA samples were sequenced using an Illumina MiSeq instrument with 250 bp paired-end reads. MG and MT samples were sequenced using Illumina HiSeq4000 and NextSeq550 instruments with paired-end 150 bp reads.

16S rRNA Bioinformatic Data Analysis

Raw 16S rRNA FASTQ sequence data were imported into DADA2^2^ for initial quality score assessment. Based on the observed quality metrics, forward reads were truncated at a length of 162 and reverse reads were truncated at a length of 231, with a maximum expected error of 1.0. Filtered reads were then subject to filtered read merging, chimera removal, denoising, and amplicon sequence variant (ASV) assignment within DADA2. Representative ASV sequences were then subject to microbial taxonomy assignment using a Naive Bayes classifier as implemented in Qiime2’s “qiime feature-classifier classify-sklearn” command^3^ with a pre-trained Silva 132 database containing 515F/806R sequences^4^. Representative sequences were also used to create a rooted phylogenetic tree using MAFFT^5^ and FastTree^6^ through Qiime 2’s “qiime phylogeny align-to-tree-mafft-fasttree” command.

Metagenomics & Metatranscriptomic Bioinformatic Data Analysis

Raw data underwent systematic quality filtration and adapter removal using Trimmomatic^7^ with parameters set to conduct a sliding window filtration at a 4-base average Q score of 20 or lower, and reads trimmed below 75 basepairs were discarded. After quality filtration, sequences were subject to the removal of all *Homo sapiens* sequences using Kraken2^8^ and the Homo sapiens NCBI reference genome. Filtered microbial reads were then subject to a follow-up annotation, which consisted of a repeated Kraken2 alignment in which microbial reads were aligned against CSI’s curated Refseq database. All annotation counts were collated into a data frame, which contains the observed sequence annotation count for each annotated taxa within each sample. All annotations first underwent count-per-million (CPM) normalization, which in turn was utilized to calculate the per-taxon CPM ratio (CPM-r) by dividing the observed experimental sample CPM measure of each taxon, by the respective negative control CPM measure of the matched taxon. All control CPM measures of 0 were converted to 1 for CPM-r calculation.

To confirm the accuracy of the observed *E. coli* annotations within the synovial fluid metatranscriptomic dataset, sequences identified as *E. coli* by Kraken2 (within those samples and the controls) were extracted and then assembled with rnaSPADES^9^ on a per sample basis. Assemblies were then aligned to the NCBI nucleotide database^10^ using BLAST^11^ with a maximum e-value of 1 * 10^-10^. The taxonomies of the best hits (based on bit score) were then reviewed to see how many of them were once more annotated as *E. coli.*

The generated annotation table as well as all associated sample metadata were merged into a *phyloseq*^12^ object within R^13^ for beta diversity analysis. Within R, the mixOmics package^14^ was utilized to conduct Partial Least Squares Discriminant Analysis (PLS-DA) of microbial taxa profiles between primary and non-primary samples. The same table was utilized for non-supervised Principal Coordinates Analysis (PCoA) of Bray Curtis distances which was generated using Qiime2. CPM-r normalized measures of select taxa of interest were visualized as one-way clustered heatmaps using the *pheatmap* R package^15^ as well as bar-plots using the ggplot2 package^16^ for 16S, MG, and MT datasets.

Kruskal-Wallis tests were conducted to identify significantly differential biomarker taxa between primary, aseptic, and infected samples using LEfSe^17^. Linear Discriminant Analysis (LDA) was used to quantify the strength of enrichment of significantly differential features (Kruskal-Wallis, p≤0.05 and log(LDA)≥ 3.0).

**References**

1. Schieffer, K. M. *et al.* The Microbial Ecosystem Distinguishes Chronically Diseased Tissue from Adjacent Tissue in the Sigmoid Colon of Chronic, Recurrent Diverticulitis Patients. *Sci. Rep.* **7**, 8467 (2017).

2. Callahan, B. J. *et al.* DADA2: High-resolution sample inference from Illumina amplicon data. *Nat. Methods* **13**, 581–583 (2016).

3. Bolyen, E. *et al.* Reproducible, interactive, scalable and extensible microbiome data science using QIIME 2. *Nat. Biotechnol.* **37**, 852–857 (2019).

4. Quast, C. *et al.* The SILVA ribosomal RNA gene database project: improved data processing and web-based tools. *Nucleic Acids Res.* **41**, D590–D596 (2013).

5. Katoh, K. & Standley, D. M. MAFFT multiple sequence alignment software version 7: improvements in performance and usability. *Mol. Biol. Evol.* **30**, 772–780 (2013).

6. Price, M. N., Dehal, P. S. & Arkin, A. P. FastTree 2 – Approximately Maximum-Likelihood Trees for Large Alignments. *PLOS ONE* **5**, e9490 (2010).

7. Bolger, A. M., Lohse, M. & Usadel, B. Trimmomatic: a flexible trimmer for Illumina sequence data. *Bioinformatics* **30**, 2114–2120 (2014).

8. Wood, D. E., Lu, J. & Langmead, B. Improved metagenomic analysis with Kraken 2. *Genome Biol.* **20**, 257 (2019).

9. Bushmanova, E., Antipov, D., Lapidus, A. & Prjibelski, A. D. rnaSPAdes: a de novo transcriptome assembler and its application to RNA-Seq data. *GigaScience* **8**, (2019).

10. NCBI Resource Coordinators. Database resources of the National Center for Biotechnology Information. *Nucleic Acids Res.* **44**, D7–D19 (2016).

11. Camacho, C. *et al.* BLAST+: architecture and applications. *BMC Bioinformatics* **10**, 421 (2009).

12. McMurdie, P. J. & Holmes, S. phyloseq: An R Package for Reproducible Interactive Analysis and Graphics of Microbiome Census Data. *PLOS ONE* **8**, e61217 (2013).

13. R Core Team. *R: A language and environment for statistical computing*. (R Foundation for Statistical Computing, 2020).

14. Rohart, F., Gautier, B., Singh, A. & Cao, K.-A. L. mixOmics: An R package for ‘omics feature selection and multiple data integration. *PLOS Comput. Biol.* **13**, e1005752 (2017).

15. Kolde, R. *pheatmap*. (2018).

16. Wickham, H. *ggplot2: Elegant Graphics for Data Analysis*. (Springer-Verlag, 2016).

17. Segata, N. *et al.* Metagenomic biomarker discovery and explanation. *Genome Biol.* **12**, R60 (2011).
